# An Obstacle Avoidance Path Planning and Evaluation Method for Intelligent Vehicles Based on the B-Spline Algorithm

**DOI:** 10.3390/s23198151

**Published:** 2023-09-28

**Authors:** Yulong Zhang, Pengwei Wang, Kaichen Cui, Hengheng Zhou, Jinshan Yang, Xiangcun Kong

**Affiliations:** School of Transportation and Vehicle Engineering, Shandong University of Technology, Zibo 255022, China; zyl16332@163.com (Y.Z.); cuikaichen19990918@163.com (K.C.); zhouhenghengai@outlook.com (H.Z.); yangjinshan53@163.com (J.Y.); 13153006626@163.com (X.K.)

**Keywords:** path planning, intelligent vehicle, B-spline, four-segment lane-changing model, comprehensive evaluation

## Abstract

To meet the real-time path planning requirements of intelligent vehicles in dynamic traffic scenarios, a path planning and evaluation method is proposed in this paper. Firstly, based on the B-spline algorithm and four-stage lane-changing theory, an obstacle avoidance path planning algorithm framework is constructed. Then, to obtain the optimal real-time path, a comprehensive real-time path evaluation mechanism that includes path safety, smoothness, and comfort is established. Finally, to verify the proposed approach, co-simulation and real vehicle testing are conducted. In the dynamic obstacle avoidance scenario simulation, the lateral acceleration, yaw angle, yaw rate, and roll angle fluctuation ranges of the ego-vehicle are ±2.39 m/s^2^, ±13.31°, ±13.26°/s, and ±0.938°, respectively. The results show that the proposed algorithm can generate real-time, available obstacle avoidance paths. And the proposed evaluation mechanism can find the optimal path for the current scenario.

## 1. Introduction

With the surge in car ownership, traffic jams and accidents are getting worse [1,2,3]. The emergence of intelligent vehicles has effectively restrained this phenomenon and brought huge benefits to society. Obstacle avoidance path planning has received extensive attention in the field of intelligent vehicles [4,5,6]. Obstacle avoidance path planning plays a crucial role in the development of intelligent vehicles. By using reasonably sound evaluation methods to determine the optimal path, intelligent vehicles are able to complete the lane-changing process in an optimal way. The main methods of obstacle avoidance path planning include the lane-changing model [7,8], the rapidly exploring random tree (RRT) [9,10,11], the artificial potential field method (APF) [12,13,14], the A* algorithm [15], the parametric curve algorithm [16,17] and so on. In existing obstacle avoidance path planning approaches, lane-changing models are widely applied. Lane-changing models are subdivided into arc lane-changing models, cosine function lane-changing models, polynomial function lane-changing models, and so on. Xu Kangjun [18] proposed an improvement scheme based on the cosine lane-changing model. The problem of the front wheel deflection angle constraint at the beginning and end of lane-changing was solved, and the continuity of the curvature of the lane-changing path was ensured. However, this method is difficult to use in complex traffic scenarios. Li Wei et al. [19] proposed a polynomial with time as a parameter to plan a lane-changing path. Reasonable obstacle avoidance paths could be generated in complex road environments. However, planning paths are difficult to modify locally. In addition, other path-planning algorithms are also widely applied. Ma et al. [20] proposed a fast RRT algorithm that improved the real-time performance and accuracy of path planning. However, the planned paths are tortuous, which has a greater impact on the stability of vehicle travel. Zhang Xiao et al. [21] proposed a Bi-RRT algorithm for obstacle avoidance path planning. Smooth path planning is implemented in a short period of time. However, there was no evaluation of the comprehensive performance of paths. ULISES et al. [22] proposed a QAPF algorithm based on Q-learning and APF. The smoothness of the path and the efficiency of the algorithm had been improved. However, vehicle dynamics constraints were ignored. Gu et al. [23] corrected the repulsive potential field of the traditional artificial potential field method and added a fuzzy control algorithm. The effectiveness and applicability of the path-planning algorithm were improved. Local optimum and target unreachable problems were difficult to completely solve. Tang et al. [24] improved A* based on the bidirectional search, a guideline, and a list of key points. Algorithm computation time was reduced. However, the dynamic path planning problem could not be solved.

The most common methods for parametric curve algorithms are Bézier and B-splines, which have the advantages of a simple calculation principle and high smoothing of planned paths. Real-time performance of path planning is met, and stability during obstacle avoidance is met [25,26]. Compared to Bézier, the B-splines algorithm has more robustness and can be locally modified by combining obstacle avoidance scenarios. The complex mathematical definition of the B-spline also determines the excellent continuity of a planned path. Therefore, B-spline is widely used in dynamic local path planning research. Zeng Dequan et al. [27] proposed a high-performance path planning method based on the cubic B-spline algorithm and a safe obstacle avoidance path generated. Berglund et al. [28] calculated B-spline curves with the goal of minimizing curvature variation and successfully planned high-speed paths. Kano et al. [29] established a link between B-spline control points and trajectory curvature. Rational trajectories were planned by setting an upper bound on curvature. Li et al. [30] combined the tentacle algorithm and the B-spline curve to plan obstacle avoidance paths. Path planning time was successfully reduced, and the real-time performance of path planning was improved. However, in terms of the specific description of the obstacle avoidance path, the B-spline algorithm is not as detailed as the lane-changing model, which leads to a complex path generation analysis process. Therefore, the B-spline algorithm needs to be further improved.

In recent years, real-time path evaluation has gained importance with the deepening of path planning research. Scholars establish evaluation methods to obtain a real-time optimal path. Qu et al. [31] used path length and smoothness as evaluation indicators to obtain the shortest feasible path that meets the kinematic constraints of vehicles. Yang et al. [32] proposed an optimal path selection method and solved the relationship between the comfort and efficiency of lane-changing. Comfort is owned by the path, and vehicles can perfectly avoid obstacles. However, multi-indicator evaluation and real-time performance are difficult to include in the establishment of evaluation mechanisms.

Therefore, in this paper, a path planning and evaluation approach for intelligent vehicles for dynamic obstacle avoidance is proposed. The main contributions of this study are summarized as follows:

- An obstacle avoidance path planning approach based on an improved B-spline algorithm is proposed. By combining the four-stage lane-changing model and adding safety constraints and dynamics constraints, the path curvature sudden change problems at obstacle avoidance start point and end point positions are solved, and obstacle avoidance path performance is improved.
- By combining real-time traffic information, a comprehensive evaluation mechanism is established. The comprehensive performance evaluation results of available paths are generated, and the real-time optimal path is obtained.
- By conducting co-simulation experiments and real vehicle tests, the availability of the proposed approach is verified.

The remainder of this paper is organized as follows: The generation process of obstacle avoidance paths is illustrated in Section 2. The multi-indicator real-time path evaluation mechanism is then developed in Section 3. Simulation and real vehicle test results are analyzed in Section 4. The research conclusions are summarized in Section 5.

## 2. Obstacle Avoidance Path Planning

Obstacle avoidance path planning is finding a path that allows vehicles to avoid obstacles safely and smoothly. This function can be realized through the given start and stop positions and constraints. The lane-changing model is employed to specifically describe obstacle avoidance paths. A detailed analysis of the obstacle avoidance process is implemented. Most existing lane-changing models have been developed based on the theoretical foundation of three-segment lane-changing [33]. The problem of a vehicle’s zero front wheel angle is not considered at the beginning of lane changing. And discontinuous curvature of lane-changing paths is caused. Later, the three-segment lane-changing theory is displaced by the four-segment lane-changing model [34]. In the process of building a four-segment lane-changing model, front wheel angle, twist angle duration, heading angle, and other parameter factors are considered. A four-segment lane-changing model solves the problems of most lane-changing models. Therefore, a four-segment lane-changing model is employed to analyze the lane-changing process in this paper.

### 2.1. Four-Segment Lane-Changing Model

**Figure 1:**
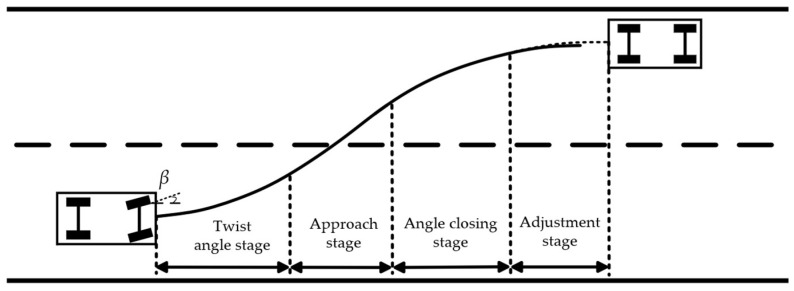
Four-segment lane-changing model.

The lane-changing process of the four-segment lane-changing model is divided into a twist angle stage, an approach stage, an angle closing stage, and an adjustment stage. In the twist angle stage, the heading angle of vehicles changes with the rotation of the steering wheel and ultimately reaches the required heading angle for lane changing. In the approach stage, vehicles maintain the heading angle at the end of the previous stage. Vehicles maintain a zero front wheel angle and move to the appropriate lane-changing position. In the angle-closing stage, the heading angle of vehicles is brought back to its initial condition with the rotation of the steering wheel while vehicles reach the target lane. After the angle-closing stage is completed, if the vehicle condition differs from ideal, the steering wheel is turned slightly to adjust the vehicle condition in the adjustment stage. The specific lane-changing model is shown in Figure 1.

Based on the analysis results of the four-segment lane-changing model, the lane-changing process for vehicles should be completed based on real-time traffic conditions. Too long or too short of a lane-changing process can have an adverse impact on traffic [35,36]. In the same scenario, the longitudinal displacement of vehicles is lengthened due to long lane changing times. Normal driving in other vehicles is impacted, and traffic capacity is reduced. If the duration of the lane-changing process is short, the lateral acceleration of vehicles will be high, which will affect comfort performance. And if the lateral acceleration of vehicles exceeds the dynamic constraints, sideslip and rollover accidents will occur, which will threaten traffic safety. Therefore, lane changing time is an important indicator to measure the reasonableness of the lane changing process.

Based on the four-segment lane-changing model, assuming that the front wheel angle of vehicles is zero in the initial state of lane-changing. By operating the steering wheel to change the front wheel angle, lane changing is implemented. According to the Ackerman steering principle [37], the instantaneous turning center of vehicles should be located on the extension line of the rear axle. And the formula for calculating the turning radius can be expressed as:

(1) $$R=\frac{L}{\tan\beta },$$

where L is the wheelbase of the vehicle and, β is the front wheel angle of the vehicle.

The initial position of intelligent vehicles for lane-changing is set as $\left(x_{0},y_{0}\right)$. The position at the end of the first stage is $\left(x_{1},y_{1}\right)$. Due to front wheel angle is a time variable. When the target heading angle is determined, the calculation formula for the time spent in the twist angle stage can be expressed as:

(2) $$\theta =\int_{0}^{t_{1}}\;\omega dt=\int_{0}^{t_{1}}\frac{v_{1}}{R}dt\;=\int_{0}^{t_{1}}\frac{v_{1}\tan\beta }{L}dt,$$

where $t_{1}$ is the duration of the twisting stage, ω is the yaw rate of the intelligent vehicle, $v_{1}$ is the speed of the intelligent vehicle. θ is the target heading angle at the end of the twist angle stage, θ is referred to as the twist angle in the following text.

In the approach stage, the travel time is defined as $t_{2}$, and the lateral distance and longitudinal distance of vehicle movement in the approach stage are respectively expressed as:

(3) $$\left\{\begin{array}{c} x=\int_{0}^{t_{2}}v_{1}\cos\theta dt\\ y=\int_{0}^{t_{2}}v_{1}\sin\theta dt \end{array}\right.,$$

Therefore, the location of vehicles is $\left(x_{1}+x,y_{1}+y\right)$ when the approach stage is completed.

The goal of the adjustment stage is to reduce the front wheel angle and heading angle to zero. However, this goal can be achieved at the angle-closing stage. Therefore, the adjustment stage can be included in the angle-closing stage. In the angle-closing stage, the heading angle is changed to the initial condition. The formula for calculating the minimum lateral distance to satisfy this requirement can be expressed as:

(4) $$d_{\min}=\frac{L-L\cos\theta }{\tan\beta_{\max}},$$

where $\beta_{\max}$ is the maximum front wheel angle.

The target position of lane-changing is defined as $\left(x_{2},y_{2}\right)$. And the end position of the angle closing stage is the target position of lane-changing, the time spent in the angle closing stage is defined as $t_{3}$. The calculation formula of $t_{3}$ can be expressed as:

(5) $$\theta =\int_{0}^{t_{3}}\frac{v_{1}\tan\beta_{\max}}{L}dt,$$

Total lane-changing time can be expressed as:

(6) $$t_{c}^{\prime }=t_{1}+t_{2}+t_{3},$$

The duration of the lane-changing process should be kept within reasonable limits. The maximum lane-changing time is defined as $t_{\max}$, and the minimum lane-changing time is defined as $t_{\min}$ in this paper. The impact of lane-changing on the traffic capacity of roads should be reduced, and demand for riding comfort should also be met. Therefore, the total lane-changing time is redefined as:

(7) $$t_{\mathrm{total}}=\left\{\begin{array}{l} t_{c}^{\prime },\;t_{\min}\leq t_{c}^{\prime }\leq t_{\max}\\ t_{\min},\;t_{c}^{\prime }<t_{\min}\\ t_{\max},\;t_{\max}<t_{c}^{\prime } \end{array}\right.,$$

### 2.2. B-Spline Algorithm

The B-spline algorithm has simple calculation principles and can generate a curve shape by combining control points with B-spline basis functions. The algorithm can be expressed as:

(8) $$P(u)=[P_{0}P_{1}P_{2}\cdots P_{n}]\left[\begin{array}{c} B_{0,k}(u)\\ B_{1,k}(u)\\ B_{2,k}(u)\\ \vdots \\ B_{n,k}(u) \end{array}\right]=\sum_{i=0}^{n}P_{i}B_{i,k}(u),$$

where $P_{i}$ is the *i*th control point, $B_{i,k}(u)$ is the *i*th k-order B-spline basis function.

The formula for the basis function is expressed as:

(9) $$B_{i,k}(u)=\left\{\begin{array}{c} \left\{\begin{array}{c} \begin{array}{l} 1,\;u_{i}\leq u\leq u_{i+1},\\ 0,\;\;otherwise, \end{array} \end{array}\;k=1\right.\\ \begin{array}{l} \frac{u-u_{i}}{u_{i+k-1}-u_{i}}B_{i,k-1}\left(u\right)+\\ \frac{u_{i+k}-u}{u_{i+k}-u_{i+1}}B_{i+1,k-1}(u),\;k\geq 2 \end{array} \end{array},\right.$$

where $u_{i}$ is the *i*th node.

Path smoothness and curvature continuity should be satisfied in the path planning process based on the B-spline algorithm. Therefore, the cubic B-spline algorithm is often used for planning. In addition, a quasi-uniform B-spline is used to further optimize the start and end positions of paths. The reason is that the first and last control points of a quasi-uniform B-spline are located at the beginning and end of paths.

According to the time period partition of the four-segment lane-changing model, B-spline control points can be set at the start and end positions of each period to generate four-segment lane-changing paths. Therefore, $P_{0}=(x_{0},y_{0})$, $P_{3}=(x_{1},y_{1})$, $P_{4}=(x_{1}+x,y_{1}+y)$, $P_{5}=(x_{2},y_{2})$. To ensure the heading angle of vehicles is zero and no sudden changes occur during the initial lane-changing. Set control point $P_{1}=(x_{0}+\frac{z}{2},y_{0})$ and $P_{2}=(x_{0}+z,y_{0})$, z is half the length of the vehicle body. After completing lane-changing for obstacle avoidance, vehicles will return to their original lane. Assuming that the head center point of the intelligent vehicle is located at $P_{4}$, and the coordinate of the rear center point of the obstacle vehicle is $\left(x_{0}^{\prime },y_{0}^{\prime }\right)$ at the same time. When the head of the intelligent vehicle exceeds one body distance from the head of the obstacle vehicle, the intelligent vehicle returns to its original lane to ensure safety in this paper. And the path of returning to the original lane is defined as symmetrical with the initial lane-changing path for obstacle avoidance. The length of the obstacle vehicle body is set as n. Therefore, the control points $P_{6}=(x_{2}+\frac{x_{0}^{\prime }-x_{2}+2z+n}{v_{1}-v_{2}},y_{2})$, $P_{7}=({2x}_{2}+\frac{x_{0}^{\prime }-x_{2}+2z+n}{v_{1}-v_{2}}\;{-x}_{1}-x,y_{1}+y)$, $P_{8}=(2x_{2}+\frac{x_{0}^{\prime }-x_{2}+2z+n}{v_{1}-v_{2}}{-x}_{1},y_{1})$, $P_{9}=(2x_{2}+\frac{x_{0}^{\prime }-x_{2}+2z+n}{v_{1}-v_{2}}{-x}_{0}-z,y_{0})$, $P_{10}=(2x_{2}+\frac{x_{0}^{\prime }-x_{2}+2z+n}{v_{1}-v_{2}}{-x}_{0}-\frac{z}{2},y_{0})$, $P_{11}=(2x_{2}+\frac{x_{0}^{\prime }-x_{2}+2z+n}{v_{1}-v_{2}}{-x}_{0},y_{0})$ are set, respectively.

Obstacle avoidance path constraints are the foundation for ensuring the generation of reasonable obstacle avoidance paths. And obstacle avoidance path constraints ensure the obstacle avoidance process is completed safely and smoothly. The constraints of the obstacle avoidance path are mainly divided into collision-free constraints and Vehicle Dynamics constraints. Combining the content of Section 2 and Section 3, it can be inferred that the generation of obstacle avoidance paths is closely related to the twist angle. Therefore, constraints on the obstacle avoidance path are equivalent to constraints on the range of the twist angle.

During the lane-changing process, the ego vehicle may collide with the wayside and the front obstacle vehicle. To solve the problem, collision-free constraints are established to ensure the safety of the vehicle.

**Figure 2:**
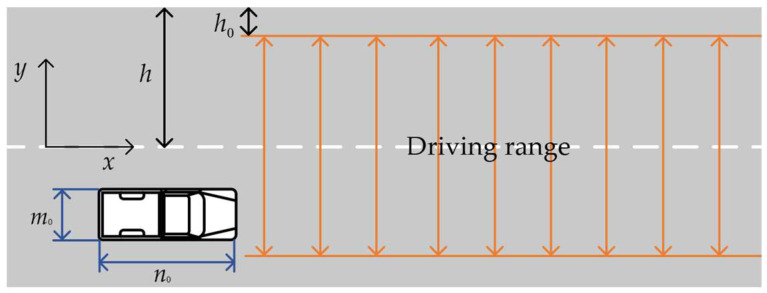
Sketch of road boundary constraints.

As shown in Figure 2. The width of the ego-vehicle is $m_{0}$. The length of the ego-vehicle is $n_{0}$. The width of the road is 2h. The real driving path of an ego-vehicle may deviate from the planned path. If road boundary constraints are formulated with actual road edge information, an ego-vehicle may collide with the road edge. Therefore, a safety distance is added between the vehicle and the road edge. The length of the safety distance is set as $h_{0}$. Road boundary constraints have been further strengthened. The range of the driving area is shown in Figure 2. The lateral range of the ego-vehicle center point can be expressed as:

(10) $$h_{0}-h+\frac{m_{0}}{2}\leq y_{\mathrm{ego}}\leq h-h_{0}-\frac{m_{0}}{2},$$

**Figure 3:**
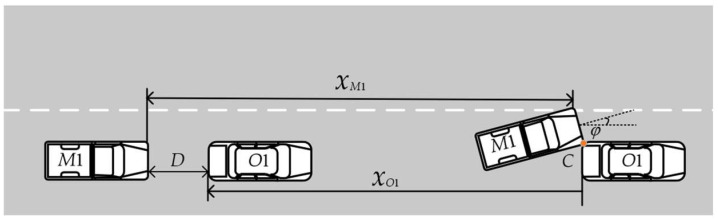
The longitudinal safety model of intelligent vehicles.

In the process of obstacle avoidance, the constraints under which the ego vehicle does not collide with the front obstacle vehicle need to be calculated. As shown in Figure 3, *C* is the critical point of the collision. The ego-vehicle is *M*1. The front obstacle vehicle is *O*1. If the ego vehicle and the front obstacle vehicle do not collide at *C*, the two vehicles will have no collision in the future. The critical condition that the ego-vehicle does not collide with the front obstacle vehicle can be expressed as:

(11) $$x_{M1}+m_{0}\sin\varphi \leq x_{O1}+D,$$

And the minimum longitudinal safety distance can be expressed as:

(12) $$\min D=\int_{0}^{t_{c}}\int_{0}^{\tau }{(a}_{1}-a_{2})d\tau dt+(v_{1}-v_{2})t_{c}+m_{0}\sin\varphi ,$$

where $v_{2}$ is the initial speed of the obstacle vehicle, $a_{1}$ is the longitudinal acceleration of the vehicle, $a_{2}$ is the longitudinal acceleration of the obstacle vehicle, $t_{c}$ is the critical collision time.

Vehicle lane-changing time is short in real traffic scenarios. Therefore, the following assumptions can be made: During the process of lane-changing intelligent vehicles to avoid obstacles, intelligent vehicles and obstacle vehicles maintain a constant speed. The critical collision time is defined as the time taken for intelligent vehicles to decelerate to the same speed as obstacle vehicles. Due to intelligent vehicles having low relative speeds to obstacle vehicles during the lane-changing process, the computational error of Equation (12) is increased. In order to ensure driving safety, this paper considers the risk level of obstacle vehicles and the relative speed of intelligent vehicles and obstacle vehicles. And the critical collision time has been redefined as:

(13) $$t_{\mathrm{cc}}=(1+\frac{n}{2(v_{1}-v_{2})})t_{c},$$

The critical collision time is used as the total lane-changing time in this paper. Therefore, intelligent vehicles complete the lane-changing process within a safe longitudinal distance to improve the safety of lane-changing.

Based on road boundary constraints and the longitudinal safety model of intelligent vehicles, the maximum and minimum twist angles can be constrained. During the lane-changing process, intelligent vehicles are expected to complete the lane-changing process as fast as possible to avoid affecting normal traffic capacity. In the twist angle stage and angle closing stage, it can be assumed that intelligent vehicles always maintain maximum front wheel angle under the current dynamic constraints. In these circumstances, the lateral displacement of intelligent vehicles increases as the twist angle increases. Under existing constraints, the maximum and minimum lateral displacement during vehicle lane-changing can be obtained. And the formula for calculating the maximum and minimum twist angle can be expressed as:

(14) $$\left\{\begin{array}{c} {2(R}_{\min}-R_{\min}\cos\theta_{\max})+v_{1}\sin\theta_{\max}t_{2}\leq \left|y_{\mathrm{ego}}\right|+h-h_{0}-\frac{m_{0}}{2}\\ {2(R}_{\min}-R_{\min}\cos\theta_{\min})+v_{1}\sin\theta_{\min}t_{2}\geq y_{M1\_O1}\\ R_{\min}=\frac{L}{\tan\beta_{\max}}\\ t_{1}=t_{3}\\ t_{2}=t_{\mathrm{cc}}-t_{1}-t_{3} \end{array}\right.,$$

where $R_{\min}$ is the minimum turning radius, $\theta_{\max}$ is the maximum twist angle, $\theta_{\min}$ is the minimum twist angle, $y_{M1\_O1}$ is the lateral displacement of the ego-vehicle from the initial state to the critical state.

Dynamic constraints are necessary to ensure the stability and safety of lane-changing vehicles. Dynamic constraints also have the function of preventing side-slip and rollover accidents in vehicles. The dynamic constraints formula can be expressed as:

(15) $$\left|{\ddot{y}}_{\mathrm{ego}}^{\prime }\right|\leq a_{y,\max}\leq 0.4g,$$

where $y_{\mathrm{ego}}^{\prime }$ is the lateral displacement of the vehicle, $a_{y,\max}$ is the maximum lateral acceleration of the vehicle, g is the acceleration of gravity.

Combined with the dynamic differential equation of the 2DOF vehicle model [38], the following formula can be obtained:

(16) $$a_{y}=\frac{{v_{1}}^{2}}{L(1+K{v_{1}}^{2})}\beta ,$$

(17) $$K=\frac{m}{L^{2}}(\frac{a}{k_{2}}-\frac{b}{k_{1}}),$$

where $a_{y}$ is the lateral acceleration of the vehicle, K is the stability factor, m is the mass of the vehicle, a is the distance from the center of mass to the front axle, b is the distance from the center of mass to the rear axle, $k_{1}$ is the cornering stiffness of the front wheel, $k_{2}$ is the cornering stiffness of the rear wheel.

Combining Formulas (16) and (17), the following formula can be obtained:

(18) $$\frac{{v_{1}}^{2}}{L(1+\;K{v_{1}}^{2})}\beta \;\leq \;0.4g,$$

Under dynamics constraints, the maximum allowable front wheel angle can be expressed as:

(19) $$\beta_{\mathrm{mot}}=0.4g\frac{L(1+K{v_{1}}^{2})}{{v_{1}}^{2}},$$

If the lane-changing process only consists of a twist angle stage and an angle closing stage, the maximum twist angle that satisfies dynamic constraints will be generated. Therefore, vehicles always maintain the maximum front wheel angle to obtain the maximum twist angle within half of the lane-changing time. And vehicles maintain a maximum reverse front wheel angle to return to the lane-changing initial state within the other half of the lane-changing time. The maximum twist angle under dynamic constraints can be expressed as:

(20) $$\theta_{\max}^{\prime }\leq \frac{v_{1}t_{\mathrm{total}}tan\beta_{\mathrm{mot}}}{2L},$$

The smaller of $\theta_{\max}^{\prime }$ with $\theta_{\max}$ is taken as the maximum allowable twist angle under the current scenario. The formula can be expressed as:

(21) $$\theta_{\mathrm{real}}=\min\left\{\theta_{\max},\theta_{\max}^{\prime }\right\},$$

## 3. Comprehensive Evaluation Mechanism

Paths with optimal performance can be obtained by path evaluation mechanisms [39]. Combined with the functions that an obstacle avoidance path should have, a comprehensive path evaluation mechanism that includes path safety, smoothness, and comfort is established. In the same scenario, feasible paths are generated by an improved B-spline algorithm and evaluated by an evaluation mechanism. The path with the smallest comprehensive evaluation result is selected as the optimal path. Due to the requirement that real-time performance should be met during the process of path planning, the number of generated paths should be minimized as much as possible to reduce computation. The sampling step length is set at 1/10 of the interval length between the maximum and the minimum twist angle in the same scenario.

**Figure 4:**
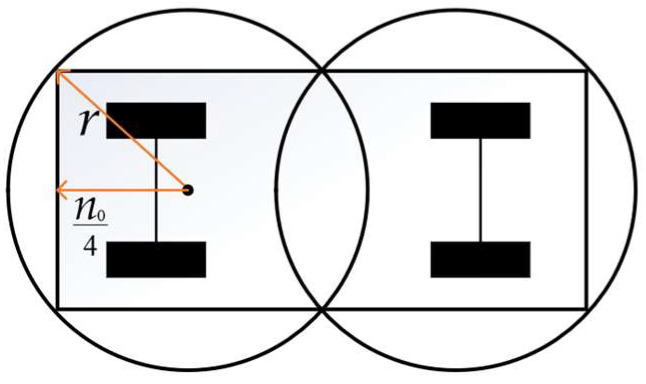
The geometric model of the intelligent vehicle.

The intelligent vehicle should have a safe distance from the front obstacle vehicle in the process of lane-changing. The bigger the ratio of this distance to the minimum longitudinal safety distance, the lower the risk level of lane-changing [40]. For a more convenient calculation of safe distance, a geometric model of the intelligent vehicle is established. Firstly, an intelligent vehicle is simplified into a rectangular model to describe its contour and dimensions. Then, circles of vehicle head and vehicle rear are established based on the rectangular model. The geometric model of an intelligent vehicle is shown in Figure 4.

A rear-end collision is the main type of collision between the intelligent vehicle and the front-end vehicle. Therefore, the safety performance of lane-changing can be determined by calculating the shortest distance between the circle of the intelligent vehicle head and the circle of the obstacle vehicle rear. The safety evaluation indicator can be expressed as:

(22) $$\min J_{d}=\frac{\min D}{d_{\min}\left(O,P\right)},$$

where $d_{\min}\left(O,P\right)$ is the shortest distance between the center of the circle of the intelligent vehicle head and the center of the circle of the obstacle vehicle rear.

The greater the change in path curvature, the worse the smoothness performance of the path. Therefore, the path smoothness can be expressed as:

(23) $$\min J_{T}=T_{\max}-T_{\min},$$

where $T_{\max}$ is the maximum curvature of the path, $T_{\min}$ is the minimum curvature of the path.

Excessive lateral and longitudinal acceleration of vehicles can cause strong discomfort among passengers [41]. The lane-changing comfort can be expressed as:

(24) $$\min J_{a}=\sqrt{a_{x,\max}^{2}+a_{y,\max}^{2}},$$

where $a_{x,max}$ is the maximum longitudinal acceleration of the vehicle.

Lane-changing safety, path smoothness, and lane-changing comfort are comprehensively considered. The comprehensive evaluation indicator can be expressed as:

(25) $$J=\sigma_{1}\min J_{d}+\sigma_{2}\min J_{T}+\sigma_{3}\min J_{a},$$

where $\sigma_{1}$, $\sigma_{2}$ and $\sigma_{3}$ are weight coefficients, and the sum of total weight coefficients is 1.

The larger the weight coefficient of the evaluation indicator, the more important the evaluation indicator is for path planning. Safety is the most important performance during vehicle driving, and comfort and path smoothness take second place. Therefore, this paper sets $\sigma_{1}=0.4$, $\sigma_{2}=0.3$, $\sigma_{3}=0.3$. Due to evaluation indicators are not in the same order of magnitude, the normalization method is used to shine a light on all data within an interval of [0, 1] in this paper.

## 4. Simulation and Real Vehicle Test

In order to verify the rationality of the path planning algorithm and evaluation approach, feasible paths in different scenarios are generated in MATLAB 2021 software. Meanwhile, based on the evaluation method, the optimal path in different scenarios is obtained. In order to verify the availability of the optimal path, MATLAB/CarSim co-simulation experiments are carried out. In addition, in order to further verify that the proposed approach can be applied to real traffic scenarios, real vehicle tests are used.

### 4.1. Co-Simulation

**Figure 5:**
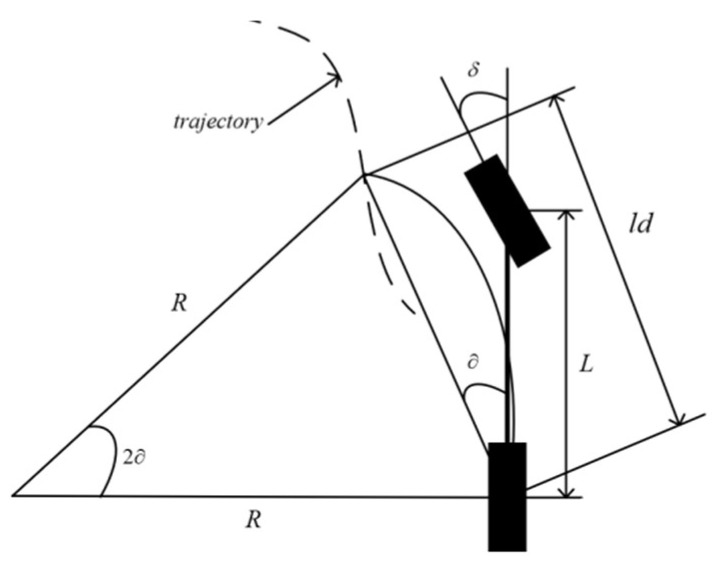
The geometric diagrammatic sketch of the pure tracking.

In co-simulation experiments, a pure tracking control model is established to achieve an intelligent vehicle tracking path [42]. The pure tracking algorithm is derived from the vehicle bicycle model [43] and the Ackerman steering principle. And the pure tracking algorithm has great robustness. The geometric diagrammatic sketch is shown in Figure 5.

In the diagrammatic sketch, *R* is the turning radius, *L* is the wheelbase of the vehicle, *ld* is the preview distance, and ∂ is the angle between the vehicle body and the preview direction.

According to the Law of sines:

(26) $$\frac{ld}{\sin(2\partial )}=\frac{R}{\sin(\frac{\pi -2\partial }{2})},$$

Then, the following formula can be obtained:

(27) $$R=\frac{ld}{2\sin(\partial )},$$

According to the Ackerman steering principle and Formula (27), the front wheel angle control quantity of the pure tracking algorithm can be expressed as:

(28) $$\delta =\alpha \tan(\frac{2L\sin(\partial )}{ld}),$$

The road is set as a two-lane road; the width of a single lane is based on the national standard of 3.75 m. The length of the ego-vehicle and obstacle vehicle is 4.79 m, the width of the ego-vehicle and obstacle vehicle is 2 m, and the wheelbase of the ego-vehicle and obstacle vehicle is 2.6 m. Ego-vehicles drive at speeds of 20 m/s and 10 m/s respectively. In their initial state, the ego vehicle and obstacle vehicle are located at the centerline of the right road. In the static traffic scenario, the abscissa of the rear of the stationary obstacle vehicle is 70 m. In the dynamic traffic scenario, the obstacle vehicle drives at speeds of 15 m/s and 3 m/s, respectively. When the obstacle vehicle speed is 15 m/s, the ego-vehicle speed is set at 20 m/s to avoid the obstacle vehicle. When the obstacle vehicle speed is 3 m/s, the ego-vehicle speed is set at 20 m/s and 10 m/s to avoid the obstacle vehicle, respectively. And when the abscissa of the rear of the dynamic obstacle vehicle is at 70 m, the ego-vehicle reaches a lane-changing position.

Under the five scenarios in this paper, the planned path number is defined as Path1-Path10 in the order of the corresponding twist angles from small to large. And the optimal path can be obtained through the comprehensive evaluation mechanism.

**Figure 6:**
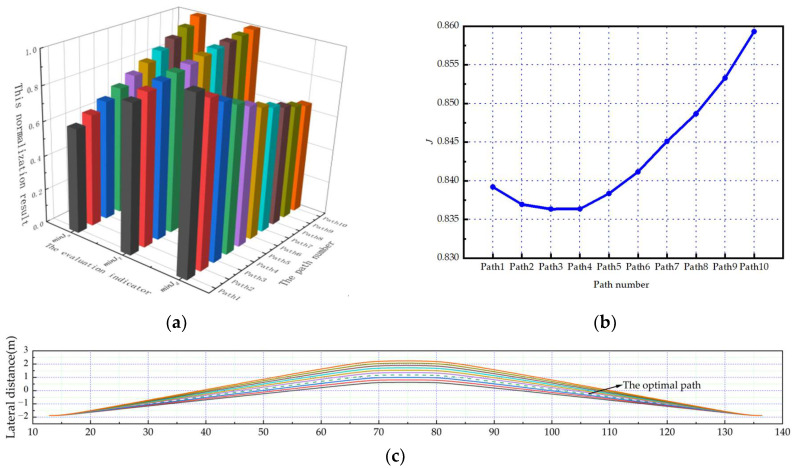
The ego-vehicle avoids stationary obstacle vehicles at 20 m/s: (**a**) normalization results of evaluation indicators; (**b**) change curve of the comprehensive evaluation indicator; (**c**) the planned path cluster.

**Figure 7:**
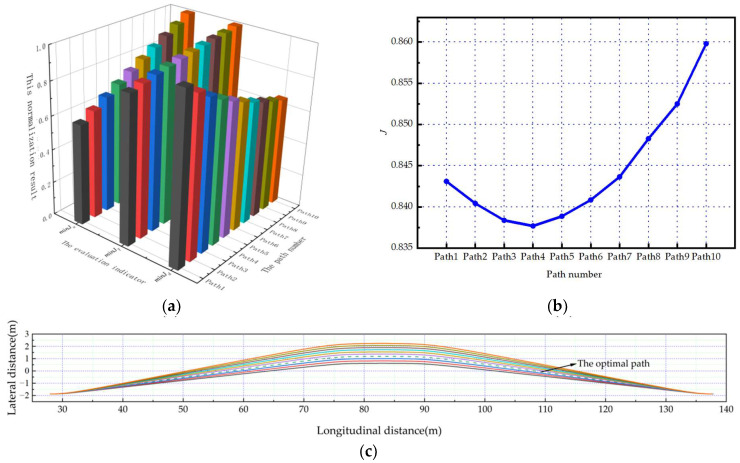
The ego-vehicle avoids low-speed obstacle vehicles at 20 m/s: (**a**) normalization results of evaluation indicators; (**b**) change curve of the comprehensive evaluation indicator; (**c**) the planned path cluster.

**Figure 8:**
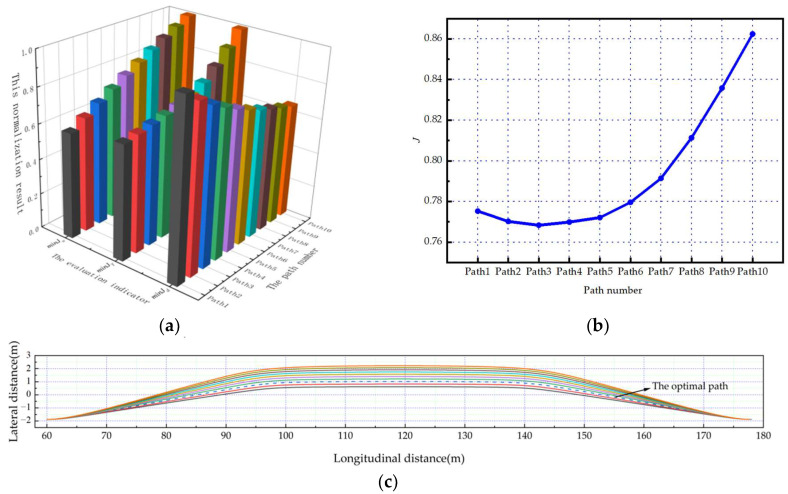
The ego-vehicle avoids a medium-speed obstacle vehicle at 20 m/s: (**a**) normalization results of evaluation indicators; (**b**) change curve of the comprehensive evaluation indicator; (**c**) the planned path cluster.

As shown in Figure 6a, Figure 7a and Figure 8a. Under the scenarios of ego-vehicles avoiding stationary obstacle vehicles, low-speed obstacle vehicles, and medium-speed obstacle vehicles at 20 m/s, $minJ_{T}$ increases as twist angle increases. And $minJ_{a}$ continuously increases as twist angle increases. This means that the smoothness of the path and the comfort of lane-changing gradually decrease as the twist angle increases. Meanwhile, $minJ_{d}$ gradually decreases as twist angle increases. This means that the safety of lane-changing increases as the twist angle increases. Based on the weight coefficients in this paper, the evaluation results of paths can be calculated. As shown in Figure 6b, Figure 7b and Figure 8b, the path corresponding to the minimum comprehensive evaluation result is the optimal path. The planned path clusters are shown in Figure 6c, Figure 7c and Figure 8c, respectively. Solid lines represent feasible paths, and dashed lines represent the optimal paths.

**Figure 9:**
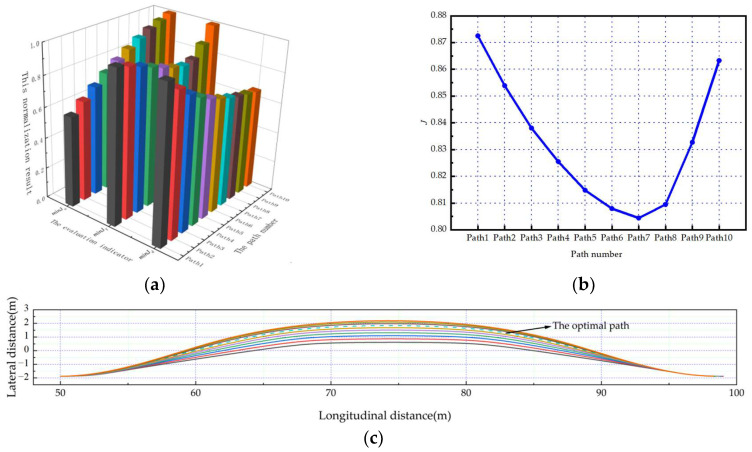
The ego-vehicle avoids stationary obstacle vehicles at 10 m/s: (**a**) normalization results of evaluation indicators; (**b**) change curve of the comprehensive evaluation indicator; (**c**) the planned path cluster.

**Figure 10:**
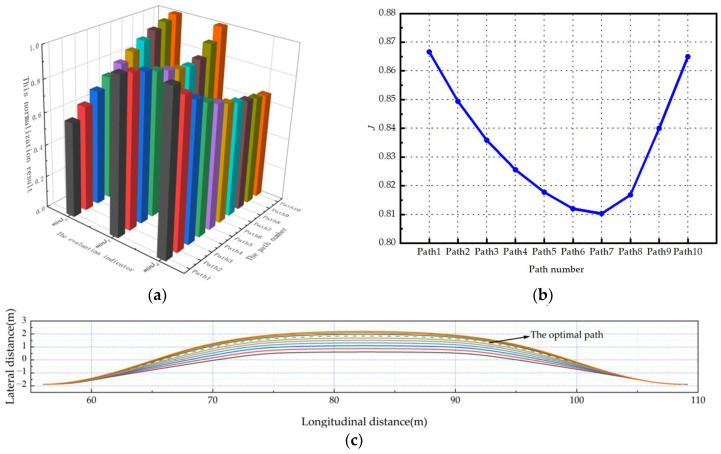

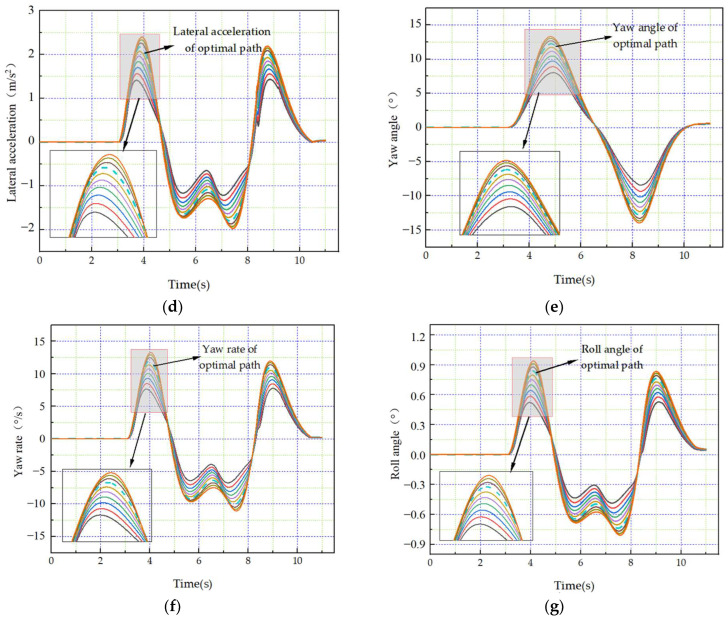
The ego-vehicle avoids a low-speed obstacle vehicle at 10 m/s: (**a**) normalization results of evaluation indicators; (**b**) change curve of the comprehensive evaluation indicator; (**c**) the planned path cluster; (**d**) lateral acceleration of the vehicle; (**e**) yaw angle; (**f**) yaw rate; (**g**) roll angle.

As shown in Figure 9a and Figure 10a. Under the scenarios of ego-vehicles avoiding stationary obstacle vehicles and low-speed obstacle vehicles at 10 m/s, $minJ_{T}$ increases as twist angle increases. This means that the smoothness of the path gradually decreases as the twist angle increases. And $minJ_{a}$ decreases first and then increases as twist angle increases. This means that the comfort of lane-changing increases first and then decreases as the twist angle increases. Meanwhile, $minJ_{d}$ gradually decreases as twist angle increases. This means that the safety of lane-changing increases as the twist angle increases. Based on the weight coefficients in this paper, the evaluation results of paths can be calculated. As shown in Figure 9b and Figure 10b, the path corresponding to the minimum comprehensive evaluation result is the optimal path. The planned path clusters are shown in Figure 9c and Figure 10c, respectively. Solid lines represent feasible paths, and dashed lines represent the optimal paths.

Under the five scenarios, the high smoothness of the path is required when ego-vehicle avoidances the low-speed obstacle vehicle at a speed of 10 m/s. Therefore, the path performance of this scenario was verified by co-simulation. Dynamic parameters of feasible paths are shown in Figure 10d–g. Dashed lines represent the dynamics parameters of the optimal paths, and solid lines represent the dynamics parameters of other paths.

As shown in Figure 10d, when an ego-vehicle drives on all feasible paths, the fluctuation range of lateral acceleration is within ± 2.39 m/s^2^, and the maximum lateral acceleration is 2.39 m/s^2^. Due to maximum lateral acceleration being less than 0.4 times that of gravitational acceleration, vehicle tires are in a linear working area. The comfort and smoothness of the vehicle are ensured. The yaw rate and yaw angle are important parameters for measuring the stability of vehicles. As shown in Figure 10e,f, the fluctuation ranges of yaw angle and yaw rate are within ±13.31° and ±13.26°/s, respectively, and the maximum yaw angle and yaw rate are 13.31° and 13.26°/s, respectively. The results show that ego-vehicles can maintain stability while driving, and tail-flick and sideslip accidents will not occur. The roll angle is an important parameter to indicate whether a rollover accident will occur. As shown in Figure 10g, the fluctuation range of the roll angle is within ±0.938°, and the maximum roll angle is 0.938°. The result shows that the possibility of vehicle rollover accidents is low when ego-vehicles track the generated paths. The optimal path not only considers stability and comfort but also emphasizes safety and performance. The optimal path makes ego-vehicles avoid obstacle vehicles safely and smoothly.

The simulation results based on five scenarios show that collision-free paths are planned based on an improved B-spline algorithm. And the ego-vehicle can smoothly return to the original lane after avoiding obstacles. The current optimal path is selected by a comprehensive evaluation mechanism. And the optimal path has a low security risk level and high smoothness. Therefore, the results demonstrate the rationality of the proposed path planning and evaluation method.

### 4.2. Real Vehicle Test

Due to the fact that simulation experiments based on virtual environments cannot fully reflect real scenarios, simulation results do not represent real conclusions. Therefore, real vehicle tests were conducted to further validate the proposed method in this paper.

**Figure 11:**
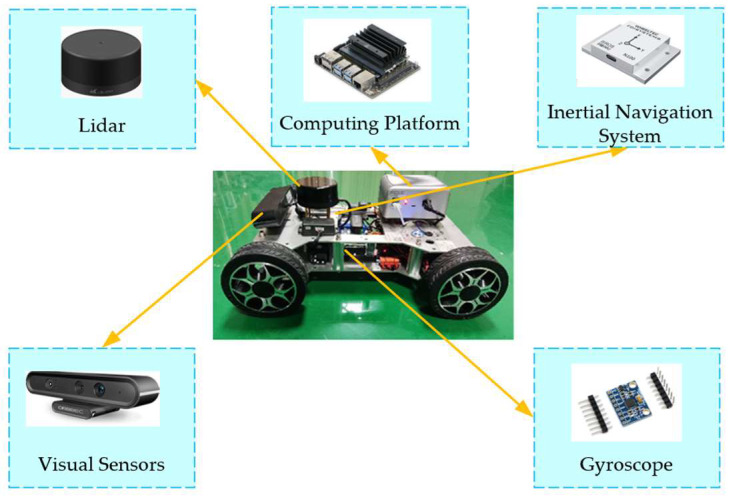
The test vehicle.

Combining the content of Section 2 and Section 3, as the width of the test vehicle increases or the width of the road decreases, vehicle feasibility is reduced. And as the speed of the test vehicle increases, the distance between the starting position of the planned path and the obstacle vehicle increases. In the experimental scenario of this paper, the size of the test vehicle conforms to the miniaturization ratio of passenger cars. The test vehicle has a wheelbase of 0.32 m and a width of 0.29 m. And the allowable fluctuation range of the front wheel angle of the test vehicle is ±23°; the requirement of tracking the miniaturization path is met. Therefore, the test vehicle can replace real passenger cars for testing. In order to obtain accurate experimental results, the experimental environment is built based on CarSim simulation scenarios and meets the miniaturization ratio. The test vehicle is equipped with Lidar, Inertial Navigation System, Mobile Chassis, Gyroscope, Visual Sensors, and Computing Platform. According to Lidar and Visual Sensors, surrounding environmental information can be perceived. According to the INS and Gyroscope, vehicle position, speed, acceleration, direction of motion, and other information can be perceived. The test vehicle is shown in Figure 11.

**Figure 12:**
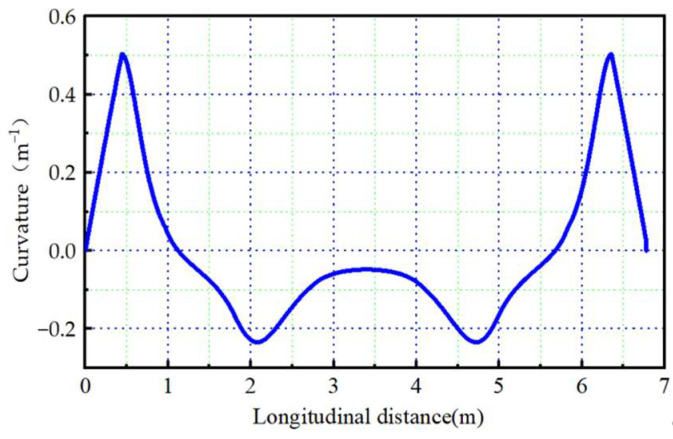
Variation curve of the miniaturized path curvature.

The scenario for an intelligent vehicle to avoid low-speed obstacles at 10 m/s was selected for the real vehicle test. Combining the test vehicle and CarSim simulation vehicle parameter information, the miniaturized path of the optimal path under this scenario can be obtained. Vehicles can only track smooth roads, and path smoothness is related to curvature. The smaller the curvature, the better the smoothness of the path [44,45]. Therefore, the curvature of the miniaturized path is calculated in this paper. The result is shown in Figure 12.

As shown in Figure 12, the curvature fluctuation range of the miniaturized path is [−0.234, 0.51], and the maximum curvature is 0.51 m^−1^. And less variation in overall curvature proves that the path is smooth.

**Figure 13:**
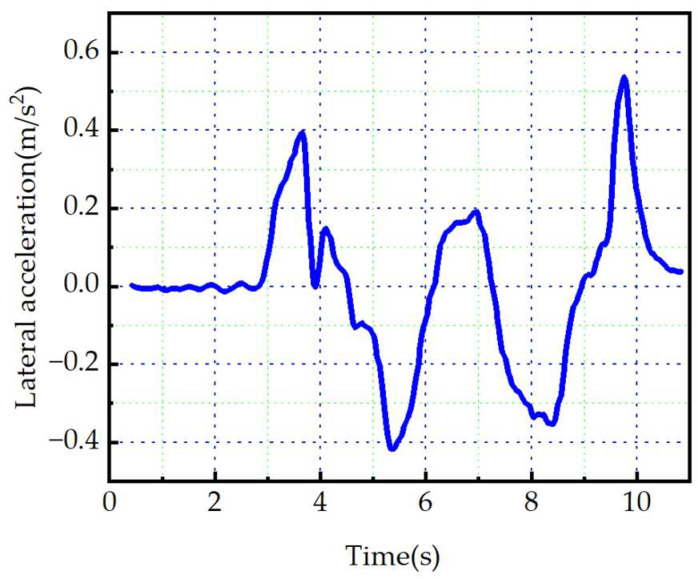
Lateral acceleration of the test vehicle.

**Figure 14:**
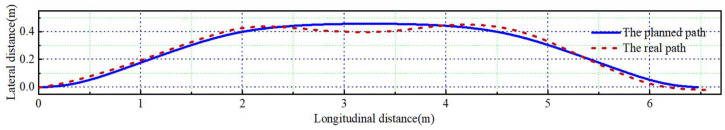
The real driving path of the test vehicle and the planned path.

Then, the test vehicle is used to track the miniaturized path. The fluctuation range of dynamic parameters of the test vehicle can be obtained, and the real driving path of the test vehicle can be obtained. Due to sensor noise in raw data, raw data are filtered based on benchmark data. As shown in Figure 13, the lateral acceleration fluctuation curve of the test vehicle is obtained after removing incorrect data. As shown in Figure 14, comparison results between the real driving path of the test vehicle and the planned path have also been obtained.

As shown in Figure 13, the lateral acceleration fluctuation range of the test vehicle is ± 0.529 m/s^2^, with a maximum value of 0.529 m/s^2^. The results show that the test vehicle can safely and smoothly avoid obstacles. As shown in Figure 14, the real driving path is in line with the planned path. The result shows that the planned path can be specifically applied to real-life scenarios.

Otherwise, it is limited by the control algorithm of the test vehicle. Deviations between the real driving path of the test vehicle and the planned path in some positions are evident.

## 5. Conclusions

To improve the ability of intelligent vehicles to avoid obstacles, an obstacle avoidance path planning and evaluation method based on the B-spline algorithm was proposed and verified. The main conclusions are as follows:

By analyzing the path planning mechanism in a dynamic traffic scenario, an obstacle avoidance algorithm framework based on B-spline and a four-segment lane-changing model is proposed. According to the proposed algorithm framework, collision-free paths are generated. To obtain the optimal real-time path, a comprehensive real-time path evaluation mechanism that includes path safety, smoothness, and comfort is established. To verify the proposed approach, co-simulation experiments and real vehicle tests are conducted. Simulation and experimental results show that the proposed algorithm framework can generate available paths for both static and moving obstacles based on traffic scenario information, and the proposed real-time evaluation mechanism can calculate the evaluation results of available paths in real-time and output the optimal path for the current scenario.

In order to cope with uncertainty in traffic scenarios, obstacle avoidance paths of ego-vehicles will be planned based on predicted paths of obstacle vehicles in the future. In addition to being limited by experimental conditions, the proposed method in this paper is verified under relatively simplified traffic scenarios. Obstacle avoidance path planning and evaluation methods will be studied in a complex environment to improve the applicability of the method.

## Data Availability

Not applicable.
